# TIMP‐2 as a Potential Indicator of Persistent Arthralgia in Chikungunya: Evidence From a Brazilian Cohort Study

**DOI:** 10.1002/jmv.70635

**Published:** 2025-10-14

**Authors:** Ana Clara Santos Costa, Anderson Felix dos Santos, Gabriela Cavalcanti Lima Albuquerque, Michelle Melgarejo da Rosa, Jamile Taniele‐Silva, Angela Luzia Branco Pinto Duarte, Maira Galdino da Rocha Pitta, André Machado de Siqueira, Amanda Pinheiro de Barros Albuquerque, Moacyr Jesus Barreto de Melo Rego

**Affiliations:** ^1^ Laboratory of Immunomodulation and New Therapeutic Approaches, Center for Research in Therapeutic Innovation – Suely Galdino (NUPIT‐SG), Center for Biosciences – CB Federal University of Pernambuco Recife Pernambuco Brazil; ^2^ Department of Nursing Nova Esperança School of Nursing (FACENE) João Pessoa Paraíba Brazil; ^3^ Head of the Rheumatology Service of HC Rheumatology Service of the Federal University of Pernambuco/Center for Health Sciences Recife Pernambuco Brazil; ^4^ Evandro Chagas National Institute of Infectious Diseases ‐ Fiocruz, Manguinhos Oswaldo Cruz Foundation (FIOCRUZ) Rio de Janeiro Rio de Janeiro Brazil; ^5^ Campus Professor Cinobelina Elvas Federal University of Piauí (UFPI) Bom Jesus Piauí Brazil

**Keywords:** arbovirus, biomarker, chronicity of musculoskeletal symptoms, extracellular matrix

## Abstract

Chikungunya virus (CHIKV) infection can trigger a sustained inflammatory response, often leading to chronic musculoskeletal pain. Matrix metalloproteinases (MMPs) and their tissue inhibitors (TIMPs) may contribute to the persistence of arthralgia through their involvement in joint tissue remodeling and degradation. This study aimed to evaluate the potential of MMPs and TIMPs as plasma biomarkers of persistent arthralgia in individuals diagnosed with Chikungunya Fever (CHIKF). A prospective cohort study was conducted using plasma samples from 133 CHIKF‐positive individuals recruited during the acute or post‐acute phases. Sociodemographic and clinical data were collected at outpatient visits. At 90 days post‐symptom onset, participants were assessed for persistent arthralgia using a self‐reported Visual Analog Scale (VAS) for joint pain and categorized into two groups: Recovered (VAS 0–3, *n* = 71) or Persistent Arthralgia (VAS 4–10, n = 62). Plasma levels of MMP‐2, MMP‐9, and TIMP‐2 were quantified using multiplex bead‐based ELISA (LegendPlex), while MMP‐1, MMP‐3, MMP‐14/MT1‐MMP, and TIMP‐1 were measured via sandwich ELISA. Plasma samples from 51 CHIKV‐negative individuals were used as controls. Statistical analyzes were performed using GraphPad Prism v9.0, IBM SPSS Statistics and Stata. Among post‐acute participants, lower MMP‐2 levels were significantly associated with recovery compared to those with persistent arthralgia and to controls. TIMP‐2 levels varied significantly across clinical outcomes, supporting its involvement in CHIKV‐induced pathology. Multivariate analysis identified swollen joints, skin rash, and elevated TIMP‐2 levels during the post‐acute phase as independent predictors of persistent arthralgia in the chronic phase. No significant differences were observed for MMP‐1, MMP‐3, MMP‐9, MMP‐14, or TIMP‐1. These findings suggest a potential role for TIMP‐2 and musculoskeletal manifestations as early indicators of chronic joint symptoms following CHIKV infection. Further studies are needed to validate these biomarkers and better understand the mechanisms underlying chronic Chikungunya‐related arthralgia across diverse populations.

## 1 Introduction

Chikungunya virus (CHIKV) is an arthropod‐borne alphavirus transmitted primarily by *Aedes* mosquito and responsible for Chikungunya fever (CHIKF), a disease characterized by acute febrile illness with prominent joint involvement [1]. Although most patients recover from the acute phase within days to weeks, approximately 30%–40% of infected individuals develop chronic musculoskeletal manifestations, most notably persistent arthralgia lasting beyond 90 days after symptom onset [2, 3, 4]. This chronic condition can lead to substantial joint pain, functional disability, absenteeism, and significant reductions in quality of life and mental well‐being [5, 6].

The transition from acute to chronic CHIKF remains incompletely understood but appears to involve prolonged or dysregulated inflammatory responses. During the acute phase, CHIKV infection activates innate immune mechanisms—particularly macrophage recruitment and activation—resulting in the release of proinflammatory cytokines and chemokines such as tumor necrosis factor‐alpha (TNF‐α), interleukin‐1β (IL‐1β), and interleukin‐6 (IL‐6) [7, 8, 9]. Persistent elevation of these mediators has been observed in some patients, suggesting a pathogenic link to long‐term joint damage [10].

One proposed mechanism involves the dysregulation of matrix metalloproteinases (MMPs), a family of proteolytic enzymes involved in extracellular matrix (ECM) remodeling. In particular, MMP‐1 and MMP‐3 have been implicated in joint tissue degradation and inflammatory arthritis [7, 11]. These enzymes are tightly regulated by tissue inhibitors of metalloproteinases (TIMPs), primarily TIMP‐1 and TIMP‐2, which serve to preserve ECM integrity and limit joint destruction. Imbalance between MMPs and TIMPs has been well documented in chronic inflammatory joint diseases and may similarly contribute to persistent arthralgia following CHIKV infection [12, 13]. Despite these insights, there remains a lack of validated biomarkers for predicting which individuals will progress to chronic CHIKF. While recent studies have highlighted inflammatory signatures that persist into the chronic phase, the precise roles of MMPs and TIMPs in CHIKV‐associated arthralgia have not been systematically investigated [14].

Therefore, the aim of this study is to investigate plasma levels of *MMP‐1, MMP‐2, MMP‐3, MMP‐9, MMP‐14, TIMP‐1, and TIMP‐2* as potential biomarkers associated with the clinical outcome of persistent arthralgia in individuals diagnosed with CHIKF. Identifying such biomarkers could improve prognostic assessment and inform the development of targeted therapeutic strategies.

## 2 Materials and Methods

### 2.1 Study Design

This was a prospective longitudinal cohort study based on clinical data and biological samples from individuals diagnosed with CHIKF. Data and samples were collected under the protocol of the multicenter project Rede de Pesquisa Clínica e Aplicada em Chikungunya (REPLICK) [15]. Participant recruitment was conducted by convenience sampling between July 2020 and August 2022. Individuals with symptoms who sought care at Hospital das Clínicas of the Federal University of Pernambuco (HC‐UFPE) or at primary healthcare units in the Recife metropolitan area within 89 days of symptom onset were invited to participate. Recruitment strategies also included public outreach via social media and local television.

Participants were referred to the Rheumatology Service at HC‐UFPE, where they were monitored during scheduled visits conducted by trained nurses and rheumatologists affiliated with the project. During these appointments, sociodemographic and clinical data were collected, and biological samples were obtained.

Sample processing, diagnostic testing (including RT‐qPCR, anti‐CHIKV IgM ELISA, and/or rapid diagnostic testing), data quality control, and subsequent experimental analyzes were performed at the Laboratory of Immunomodulation and New Therapeutic Approaches (LINAT), part of the Center for Research in Therapeutic Innovation—Suely Galdino (NUPIT‐SG), both located at UFPE.

All participants provided written informed consent in accordance with national ethical guidelines for research involving human subjects. The study protocol was approved by the Ethics Committee of the Federal University of Pernambuco (approval no. 3.555.583; CAAE: 07936919.8.2010.5208). This study was conducted and reported in accordance with the STROBE (Strengthening the Reporting of Observational Studies in Epidemiology) guidelines for observational research.

### 2.2 Inclusion and Exclusion Criteria of the Study Population

Participants were included in the study based on a confirmed diagnosis of CHIKF, determined by one or more of the following diagnostic methods: RT‐qPCR [IBMP Biomol ZDC (Zika, Dengue and Chikungunya)]—Curitiba, Brazil), anti‐CHIKV IgM ELISA (Thermo Fisher Scientific—Waltham, USA), and/or rapid diagnostic test (ZDC Biomanguinhos—Rio de Janeiro, Brazil). Diagnosis occurred during either the acute or post‐acute phase of illness. The inclusion criteria were:

(I) age ≥ 18 years;

(II) provision of signed informed consent;

(III) laboratory‐confirmed CHIKF diagnosis (via RT‐qPCR, IgM ELISA, and/or rapid test); and

(IV) availability of complete clinical data and plasma samples at both (a) the first visit during the acute [1–14 days post‐symptons onset (DPSO)]/post‐acute phase (15–89 DPSO) and (b) the first visit during the chronic phase (≥ 90 DPSO).

Exclusion criteria included the absence of both clinical data and biological samples at either follow‐up visit. Follow‐up losses were attributed to changes in participant residence, voluntary withdrawal, or missed scheduled visits.

Of the 886 individuals with suspected CHIKF initially recruited by the REPLICK project, 115 tested negative for CHIKV. An additional 439 were excluded due to incomplete clinical data and/or the absence of plasma samples in the biobank. After applying the eligibility criteria, a total of 133 CHIKF‐positive individuals were included in this study. Additionally, we included 51 CHIKF‐negative individuals (ELISA IgM and IgG), matched for sex and age, as a control group.

### 2.3 Data Collection During Clinical Follow‐Ups

Staff medical professionals collected sociodemographic and clinical data (pre‐existing comorbidities and clinical symptoms presented during the acute and post‐acute phases of the disease) using semistructured questionnaires during clinical follow‐up. A self‐reported visual analog scale (VAS), ranging from 0 to 10, was used to evaluate joint pain, fatigue, and overall disease activity. Disease activity was measured using the Clinical Disease Activity Index (CDAI), which evaluates 28 painful and swollen joints, along with patient and physician global assessment on VAS for estimating disease activity. The CDAI has range from 0 to 76 and categorized as follows: remission (≤ 2.8), low disease activity (> 2.8 and ≤ 10), moderate disease activity (> 10 and ≤ 22), and high disease activity (> 22) [16].

Participants were followed through scheduled visits, with the initial assessment conducted at enrollment and subsequent follow‐ups occurring between 21 and 90 days after recruitment. At each visit, clinical data and biological samples were collected [15]. All data were anonymized and recorded using REDCap (Research Electronic Data Capture), with each participant assigned a unique identification code.

### 2.4 Clinical Outcome Classification Criteria in the Chronic Phase

During the first visit in the chronic phase (≥ 90 days post‐symptom onset), participants were classified into two groups based on self‐reported joint pain using the VAS in response to the question: “How much joint pain have you had in the past week?” Participants with VAS scores between 0 and 3 were classified as Recovered (*n* = 71), while those scoring between 4 and 10 were categorized as having Persistent Arthralgia (*n* = 62).

### 2.5 Sandwich ELISA

Plasma concentrations of MMP‐1, MMP‐3, MMP‐14/MT‐1, and TIMP‐1 (R&D Systems, Minneapolis, USA) were quantified in 133 plasma samples collected during the acute and post‐acute phases, following the manufacturer′s instructions. The dilution factors were as follows: MMP‐1 and MMP‐3 (1:8), MMP‐14/MT‐1 (1:2), and TIMP‐1 (1:150).

### 2.6 Multiplex ELISA

MMP‐2, MMP‐9, and TIMP‐2 levels were analyzed using the LegendPlex multiplex technology with antibody‐coated beads (BioLegend, San Diego, USA), employing the Custom Human 3‐plex Panel kit (Item No: 900004902) in 75 plasma samples collected during the acute and post‐acute phases. Data analysis was performed using the LEGENDplex Qognit data analysis software.

### 2.7 Statistical Analysis

Statistical analyzes were performed using GraphPad Prism version 9, IBM SPSS Statistics version 25 and Stata version 18 (StataCorp LLC, College Station, TX, USA). Data normality was assessed using the Shapiro‐Wilk test. Group comparisons were conducted using Student′s t‐test, Mann‐Whitney U test, one‐way ANOVA, or Kruskal‐Wallis test with appropriate post hoc analyzes. Categorical variables were analyzed using Chi‐square or Fisher′s exact test.

Receiver operating characteristic (ROC) curves were generated to assess diagnostic accuracy, with the Youden Index applied to determine optimal cut‐off values. Binary and multivariate logistic regression models (Backward Stepwise with robust variance) were used to identify predictors of persistent arthralgia. Variable selection was based on statistical significance (*p* < 0.20) and clinical relevance to musculoskeletal outcomes. A *p*‐value < 0.05 was considered statistically significant (see Supporting Information Table S1).

## 3 Results

### 3.1 Sociodemographic and Clinical Characterization of the Study Population

Of the 886 individuals with suspected CHIK recruited from the multicenter REPLICK study—Recife Center, 554 were excluded for not meeting the study′s inclusion criteria. Among the eligible participants, 133 CHIK+ were selected for investigations. Based on clinical outcomes in the chronic phase, CHIK+ participants (*n* = 133; 100%) were categorized into two groups: Recovered (*n* = 71; 53.38%) and Persistent Arthralgia (*n* = 62; 46.62%), depending on the persistence of arthralgia. The majority of participants (*n* = 109; 82%) were enrolled during the post‐acute phase, with a mean interval of 36.26 ± 21.22 days from symptom onset. The mean age of the overall cohort was 52.49 ± 14.73 years, with a predominance of biological females (*n* = 102; 76.7%) (Table 1). In the Control group, the mean age was 45.66 ± 10.27 years (range: 35–69 years), with a similar sex distribution—females (*n* = 37; 72.5%) and males (*n* = 14; 27.5%).

**Table 1:** – Recruitment and sociodemographic characteristics of the cohort of individuals diagnosed with CHIK.

Factors			CHIK +		Recovered		Persistent Arthralgia	
			*N*	%	*N*	%	*N*	%
			133	100	71	53.38	62	46.62
Enrollment	Days post‐symptoms onset (DPSO)	*Mean. DP (Min–Max)*	36.26 ± 21.22	(2–86)	41.52 ± 20.73	(2–86)	30.24 ± 20.31	(3–77)
	Diagnostic method	*Serology*	72	54.1	41	57.7	31	50
		*RT‐qPCR*	16	12	3	4,2	13	21
		*Rapid Test*	45	33.8	27	38	18	29
	CHIK phase	*Acute*	24	18	5	7	19	30.6
		*Post‐acute*	109	82	66	93	43	69.4
Sociodemografic	Age	*Mean. DP (Min–Max)*	52.49 ± 14.73	(19–84)	53.18 ± 16.18	(19–84)	51.69 ± 12.97	(20–76)
	*Biological sex*	*Female*	102	76.7	50	70.4	52	83.9
		*Male*	31	23.3	21	29.6	10	16.1

Abbreviation: SD, standard deviation.

### 3.2 Comorbidities and Clinical Manifestations

**Figure 1:**
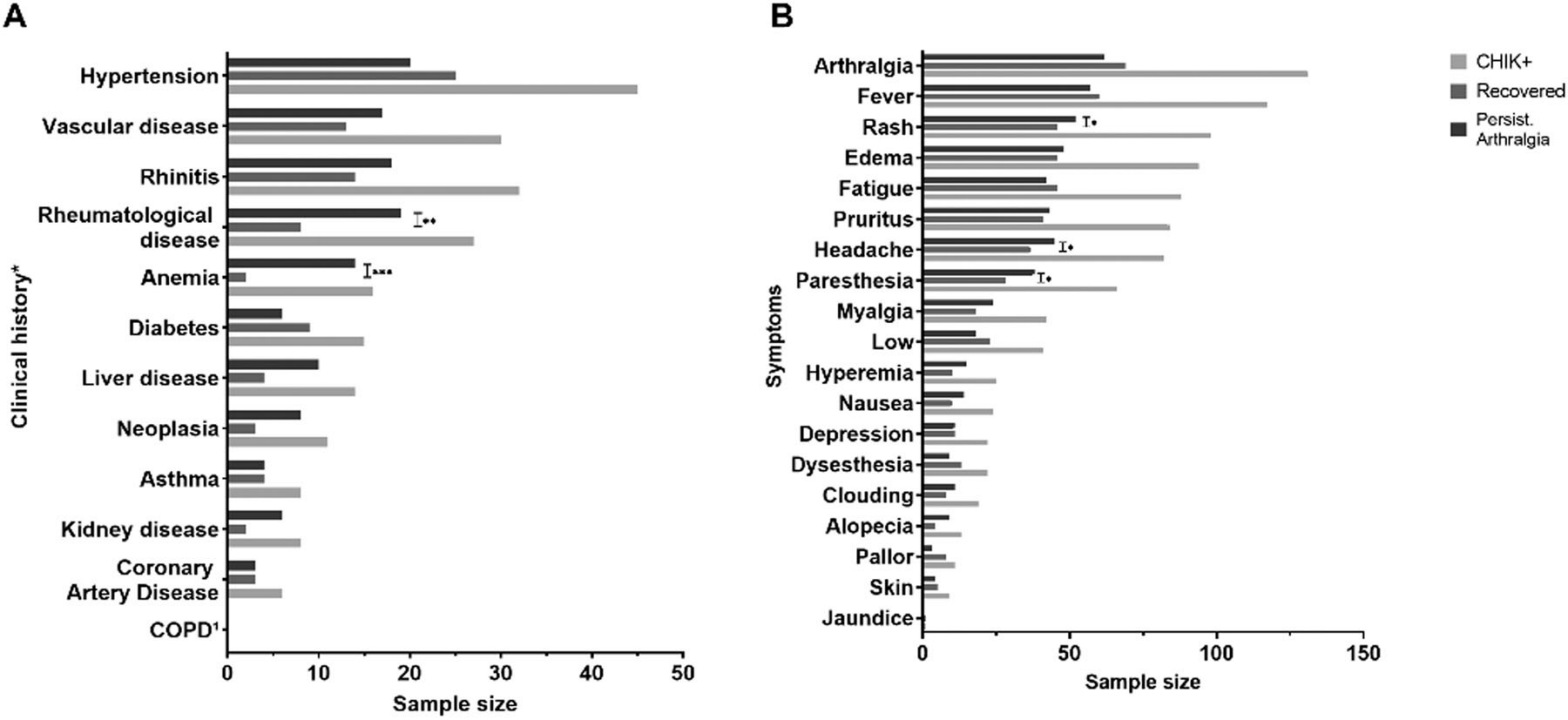
Comparison among CHIK+ groups (*n* = 133), Recovered (*n* = 71; 53.38%), and Arthralgia Persistent (*n* = 62; 46.62%) regarding clinical data collected during the acute and post‐acute phases of CHIK. (A) Clinical history (pre‐existing comorbidities). (B) Clinical symptoms. Light gray: CHIK+ group; Dark gray: Recovered group; Black: Chronic group. Data are presented as the number of individuals. *Only positive values for clinical history and symptoms at the time of recruitment were reported. 1 Chronic obstructive pulmonary disease. Statistical representation of *p*‐values: *p* ≤ 0.05 → *; *p* ≤ 0.01 → **; *p* ≤ 0.001 ***.

The most frequently reported comorbidities during the acute or post‐acute phases in the CHIK‐positive population included hypertension (*n* = 45; 33.8%), rhinitis (*n* = 32; 24.1%), and vascular disease (*n* = 30; 22.6%) (Figure 1A). The most prevalent clinical symptoms during the early phase of infection were arthralgia (n = 131; 98.5%), fever (*n* = 117; 88%), skin rash (*n* = 98; 73.7%), edema (*n* = 94; 70.7%), fatigue (n = 88; 66.2%), pruritus (*n* = 84; 63.2%), and headache (*n* = 82; 61.7%) (Figure 1B).

The chi‐square association test between the Recovered and Persistent Arthralgia groups revealed that individuals who developed persistent arthralgia during the chronic phase reported significantly higher rates of pre‐existing rheumatologic disease (*n* = 19; 30.6%; *p* = 0.008), and anemia (*n* = 14; 22.6%; *p* < 0.001) (Figure 1A). Chronic CHIKF individuals also reported significantly more frequently skin rash (*n* = 52; 83.9%; *p* = 0.013), headache (*n* = 45; 72.6%; *p* = 0.015), and paresthesia (*n *= 38; 61.3%; *p* = 0.012) (Figure 1B).

### 3.3 Rheumatologic Findings and Joint Involvement

Rheumatologic parameters (Table 2) indicated that individuals exhibiting moderate to high disease activity during the acute or post‐acute phases were significantly more likely to develop persistent arthralgia (*p* = 0.002). This was corroborated by the higher mean CDAI scores observed in the Chronic group compared to the Recovered group (27.79 ± 13.87 [range: 4–64] vs. 17.27 ± 11.48 [range: 0–59]; *p* < 0.001).

**Table 2:** Frequency of musculoskeletal disorders during the acute and post‐acute phases of CHIK according to clinical outcomes (*n* = 133).

Factors			CHIK +		Recovered		Persistent Arthralgia	
			*N*	%	*N*	%	*N*	%
			133	100	71	53.38	62	46.62
Rheumatological measurements	Disease activity category*	*Remission (< 2.8)*	5	3.8	5	7	0	0
		Low (≤ 10)	21	15.8	16	22.5	5	8.1
		Moderate (> 10 e ≤ 22)	49	36.8	27	38	22	35.5
		*High (> 22)*	58	43.6	23	32.4	35	56.5
	Clinical disease activity index (CDAI)	*Mean. DP (Min–Max)*	36.26 ± 21.22	(2–64)	17.27 ± 11.48	(0–59)	27.79 ± 13.87	(4–64)
	Number of painful joints	*Mean. DP (Min–Max)*	9.00 ± 8.25	(0–28)	8.97 ± 7.49	(0–28)	9.03 ± 9.10	(0–22)
	Number of swollen joints	*Mean. DP (Min–Max)*	2.16 ± 3.54	(0–22)	1.92 ± 2.87	(0–10)	2.44 ± 4.26	(0–22)

Abbreviation: SD, standard deviation.

* Classification based on the Clinical Disease Activity Index (CDAI).

**Figure 2:**
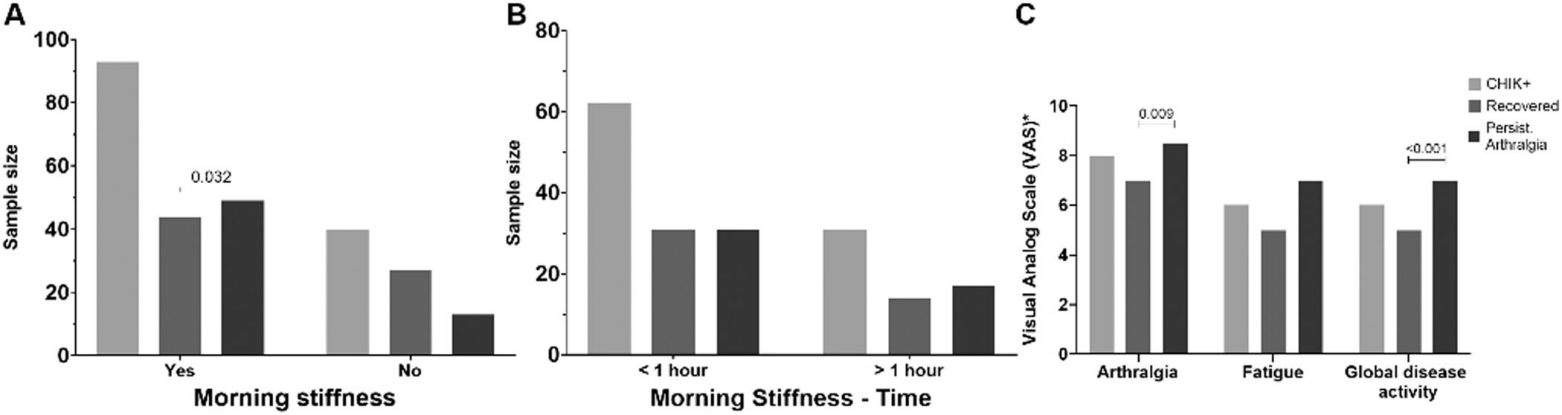
Musculoskeletal symptom involvement in CHIK+ individuals and comparison between Recovered and Chronic groups during the acute and post‐acute phases of the disease. (A) Morning stiffness; (B) Duration of morning stiffness dichotomized as greater or less than 1 h; (C) Visual Analog Scale (VAS) for arthralgia, fatigue, and overall disease activity. Groups: CHIK+ (light gray), Recovered (medium gray), and Persistent Arthralgia (black). Bars represent the absolute frequency of individuals with or without the reported symptoms. In graphs B and C, only individuals reporting the symptoms are included. *Visual Analog Scale (VAS) values are self‐reported on a 0 to 10 scale. Pearson′s chi‐square test was performed.

The number of tender and swollen joints remained elevated across all groups (CHIK + , Recovered, and Chronic). However, a trend toward reduced joint swelling was noted over time, suggesting that CHIKV may exert sustained effects on joint pain pathways independent of overt edema (Table 2). Moreover, persistent arthralgia was significantly associated with the presence of morning stiffness in early stages (*p* = 0.032; Figure 2A), although the duration of this symptom did not reach statistical significance (*p* = 0.066). Notably, most CHIK+ individuals reported morning stiffness lasting less than 1 h, suggesting a predominantly mechanical rather than inflammatory etiology (Figure 2B).

### 3.4 Pain Perception and Disease Activity

Self‐reported Visual Analog Scale (VAS) scores for joint pain and overall disease activity during early infection were significantly higher among individuals who later developed chronic arthralgia. Specifically, higher early‐phase scores for joint pain (*p* = 0.009) and overall disease activity (*p* < 0.001) were associated with persistent musculoskeletal symptoms in the chronic phase (Figure 2C).

#### 3.4.1 Soluble Mediators TIMP‐2 and MMP‐2 are Associated With the Clinical Evolution of Chikungunya

**Figure 3:**
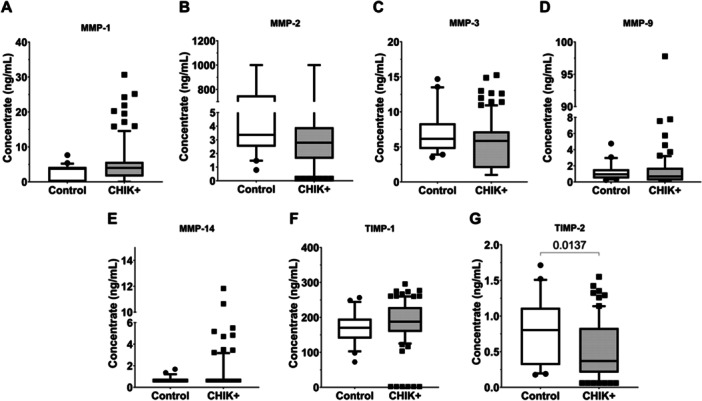
– Plasma levels of soluble mediators MMPs and TIMPs in Control and CHIK+ groups. (A) MMP‐1; (B) MMP‐2; (C) MMP‐3; (D) MMP‐9; (E) MMP‐14; (F) TIMP‐1; (G) TIMP‐2. Statistical significance was defined as *p* ≤ 0.05.

Comparative analysis between control individuals and CHIKV‐positive (CHIK + ) patients revealed that plasma levels of TIMP‐2 (Figure 3G) were significantly lower in CHIK+ subjects compared to controls (*p* = 0.013). No statistically significant differences were observed between groups for the matrix metalloproteinases (MMPs)—MMP‐1, MMP‐2, MMP‐3, MMP‐9, and MMP‐14—or for TIMP‐1.

**Figure 4:**
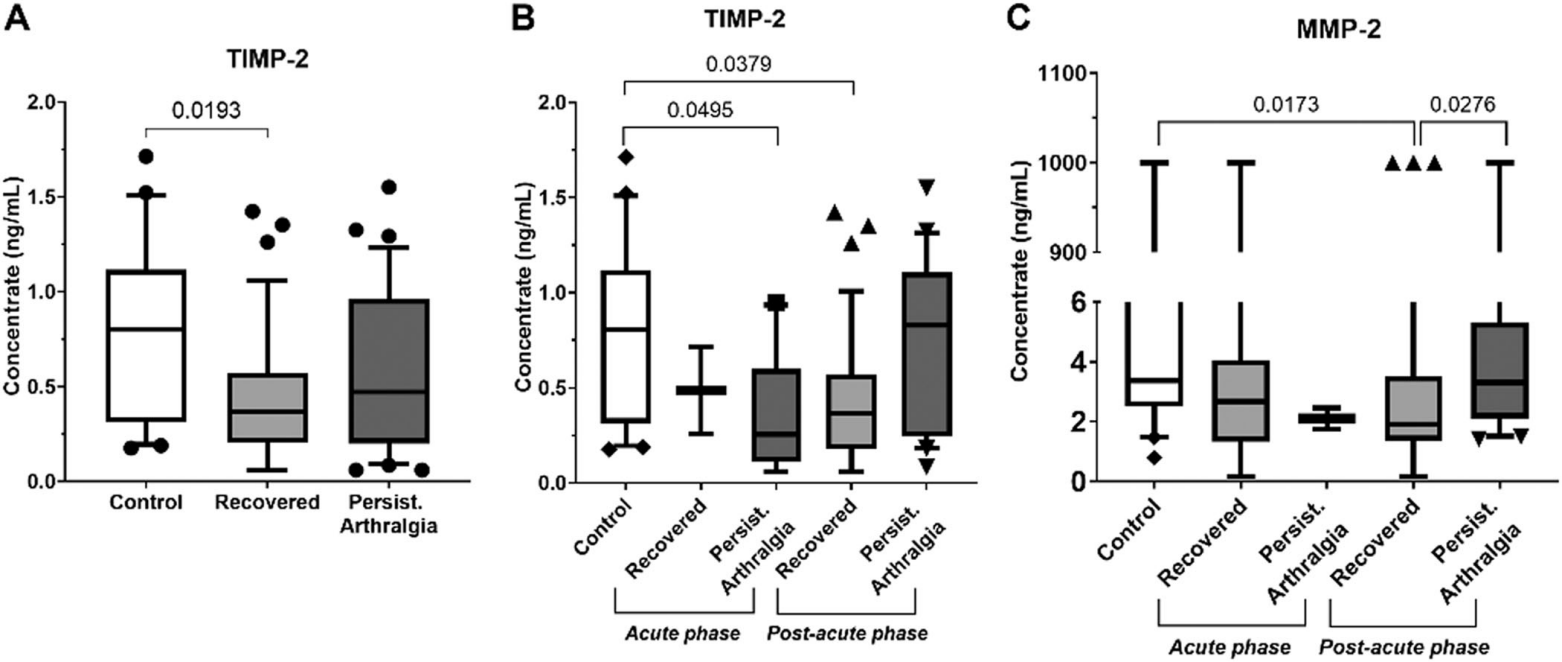
CHIKV impact on TIMP‐2 and MMP‐2 mediators considering CHIKF clinical phases and clinical outcomes of resolution (Recovered group) or persistent arthralgia after 90 DPSO compared to the Control group. (A) Plasma TIMP‐2 levels among Control, Recovered, and Persistent Arthralgia groups; (B) Plasma TIMP‐2 levels in Control and individuals recruited during acute and post‐acute phases, classified according to clinical outcomes; (C) Plasma MMP‐2 levels in Control and individuals recruited during acute and post‐acute phases, classified according to clinical outcomes. *DPSO, days post‐symptom onset. Acute phase = 1–14 DPSO. Post‐acute phase = 15–89 DPSO. Statistical significance was set at *p* ≤ 0.05.

When evaluating clinical outcomes in the chronic phase, individuals who had recovered by 90 DPSO still exhibited reduced TIMP‐2 levels compared to the control group (*p* = 0.0193) (Figure 4A). No significant differences were observed for the other soluble analytes when comparing Control, Recovered, and Persistent Arthralgia groups (Supporting Information Figure S1).

Stratifying by recruitment phase, individuals enrolled during the acute phase who went on to develop persistent arthralgia beyond 90 DPSO showed lower plasma TIMP‐2 levels compared to controls. Similarly, among those recruited in the post‐acute phase, individuals who recovered also had significantly reduced TIMP‐2 levels relative to controls (*p* = 0.037; Figure 4B). In addition, among individuals recruited during the post‐acute phase who recovered clinically, plasma levels of MMP‐2 were lower than those observed in the control group (*p* = 0.017). These individuals also exhibited significantly lower MMP‐2 levels compared to those in the same recruitment phase who developed persistent arthralgia after 90 DPSO (*p* = 0.027; Figure 4C).

Despite these associations, the mediators analyzed did not demonstrate adequate performance as predictive biomarkers of clinical outcome (Supporting Information Figure S2). No other significant differences were detected for the remaining soluble mediators across the experimental groups.

#### 3.4.2 Musculoskeletal Factors in the Post‐Acute Phase Associated With Persistent Arthralgia

Building on previous findings, we investigated whether plasma levels of MMPs and TIMPs measured during the post‐acute phase were independently associated with the development of persistent arthralgia in the chronic phase. To this end, a multivariate logistic regression analysis was conducted to identify predictors of chronicity.

The logistic regression revealed that the presence of skin rash (OR = 14.39; 95% CI: 3.29–63.00; *p* < 0.001) and swollen joints (OR = 10.87; 95% CI: 2.45–48.12; *p* = 0.002) during the post‐acute phase were independently associated with the likelihood of developing persistent arthralgia. Furthermore, each 1 ng/mL increase in plasma TIMP‐2 levels during the post‐acute phase was associated with 2.5‐fold higher odds of persistent arthralgia in the chronic phase (OR = 2.50; 95% CI: 1.24–5.06; *p* = 0.010).

**Figure 5:**
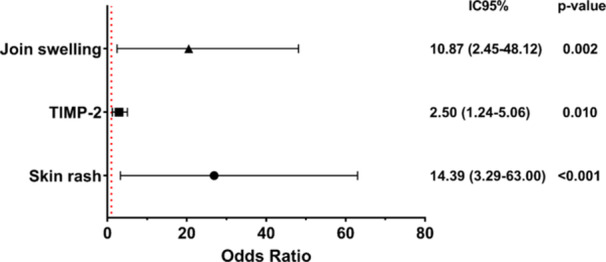
Final model of factors associated with chronic clinical outcomes among individuals recruited during the post‐acute phase of Chikungunya (*n* = 109). CI, confidence Interval; OR, odds ratio. The red dashed line represents the null effect (OR = 1). ORs were estimated by multivariate logistic regression, adjusted for potential confounders. Statistical significance was set at *p* < 0.05.

The final model was statistically significant (*p* < 0.001) and yielded a Pseudo R² of 0.3596, indicating that the musculoskeletal symptoms and TIMP‐2 levels together explained approximately 35% of the variance in the chronicity outcome (Figure 5).

## 4 Discussion

The exacerbated production of proinflammatory cytokines and chemokines—driven by Th1 and Th2 cell activation during CHIKV infection—plays a central role in the progression and persistence of clinical symptoms [8, 17]. In the chronic phase, elevated levels of MMP‐1 and MMP‐3, along with cytokines such as TNF‐α, IL‐1β, IL‐6, IL‐8, and MCP‐1, have consistently been associated with persistent arthralgia when compared to recovered individuals and healthy controls [18, 19]. These findings underscore the importance of characterizing the temporal behavior of inflammatory mediators to better understand their role in the musculoskeletal sequelae of CHIKV infection.

Chronic arthritis following CHIKV infection shares clinical and pathogenic similarities with osteoarthritis (OA), influenced by factors such as age, obesity, and joint injury [20]. Key mediators like MMP‐1, MMP‐3, MMP‐9, and MMP‐13 are central to extracellular matrix degradation and have been associated with cartilage damage in animal models and in patients with OA [21, 22]. Elevated plasma levels of MMP‐3 and MMP‐9 have been associated with disease severity, and synovial fibroblasts may contribute to OA progression through cellular senescence and increased MMP‐3 expression [23]. In the context of CHIKV infection, the dysregulated expression of these MMPs may promote persistent inflammation and tissue remodeling, contributing to the chronicity of joint symptoms [19]. Therefore, this study is among the first to evaluate the association between plasma levels of MMPs, their endogenous inhibitors (TIMPs), and the chronicity of musculoskeletal symptoms in individuals with CHIKF.

Our multivariate analysis revealed that rash, lower limb edema, and elevated TIMP‐2 levels during the post‐acute phase were significantly associated with the progression to chronic arthralgia. These findings suggest that both clinical manifestations and TIMP‐2 in the post‐acute phase may serve as indicators of poor prognosis.

Emerging evidence has shown that TIMP‐2 plays a dual role in both modulating extracellular matrix turnover and promoting tissue regeneration by stimulating collagen synthesis and increasing markers such as PIICP and PIIANP, which are key to cartilage repair in osteoarthritis [21, 24]. Despite this potential regenerative function, in our cohort, elevated TIMP‐2 levels were associated with persistent arthralgia, suggesting that its upregulation may reflect ongoing tissue remodeling in response to unresolved inflammation. However, our diagnostic performance analysis indicated that TIMP‐2 alone had limited prognostic utility, potentially due to sample size constraints—an acknowledged limitation that may affect statistical power and generalizability [25].

Additional symptoms such as headache, paresthesia, morning stiffness, and high disease activity scores were also linked to the persistence of musculoskeletal complaints. These results are consistent with findings from diverse epidemiological settings. In Puerto Rico, Medina‐Cintrón et al. [26] reported that myalgia, arthralgia, and moderate disease activity in the early phases predicted CHIKV‐related chronic arthritis. Similarly, a longitudinal study in Colombia found that rash and headache were common during the acute phase, while paresthesia persisted in up to 72% of patients even 7 years postinfection [14]. Morning stiffness was more prevalent among individuals with post‐CHIKV chronic rheumatism. Collectively, these findings support the hypothesis that specific symptoms in the early clinical course may directly influence long‐term functional outcomes and quality of life [27].

We also observed that pre‐existing immunoregulatory conditions, such as rheumatologic diseases and anemia, were associated with chronic arthralgia. Patients with prior rheumatologic diagnoses may experience impaired resolution of inflammation, potentially exacerbated by chronic use of DMARDs, which can lead to therapeutic resistance or suboptimal immunosuppressive effects [28]. Anemia may reflect a chronic inflammatory state mediated by IL‐6 and hepcidin or result from virus‐induced hematopoietic dysfunction [29, 30]. Although rarely described, anemia has been reported as an atypical manifestation of CHIKF, along with leukopenia, hepatic enzyme elevations, renal function alterations, and hypocalcemia [31, 32].

Previous studies have also shown that severe pain during the acute phase is a strong predictor of chronic disease progression [2, 5, 33] Gérardin et al., 2013 [34]. In our study, individuals who recovered within 90 days post symptom onset displayed significantly lower plasma MMP‐2 levels compared to both controls and individuals with persistent arthralgia. This suggests that CHIKV may modulate MMP‐2 expression in a phase‐dependent manner, with its downregulation potentially marking a favorable clinical trajectory. Although MMP‐2 overexpression has been implicated in fibroblast‐like synoviocyte migration and joint damage in chronic inflammatory diseases [35], its specific role in CHIKF remains to be elucidated.

This study has limitations, including a relatively small sample size, reliance on self‐reported symptoms and comorbidities, and an observational design that may be subject to confounding factors, such as undocumented health conditions or unmeasured medication use. Nevertheless, our findings contribute to the understanding of the inflammatory landscape in CHIKF and highlight potential molecular and clinical predictors of chronic musculoskeletal outcomes.

In conclusion, this study offers novel insights into the involvement of MMPs and TIMPs in the pathogenesis and progression of post‐CHIKV chronic arthralgia. Our data provide a foundation for future multicenter, longitudinal studies aiming to validate these molecules as prognostic biomarkers and to develop personalized therapeutic strategies to prevent chronic rheumatologic complications in CHIKF patients.

## 5 Conclusion

This study identified that swollen joints, rash, and lower plasma levels of TIMP‐2 during the post‐acute phase are associated with a higher risk of developing persistent arthralgia in chronic CHIKF, alongside reduced MMP‐2 secretion. These findings suggest a potential role for TIMP‐2 and MMP‐2 in the pathophysiology of post‐CHIKV musculoskeletal symptoms. Further multicenter and longitudinal studies are warranted to validate these associations and explore their relevance as prognostic biomarkers across diverse populations.

## Author Contributions

**Ana Clara Santos Costa:** writing – review and editing, writing – original draft, methodology, investigation, formal analysis, data curation, conceptualization. **Anderson Felix dos Santos:** writing – review and editing, methodology, investigation, formal analysis, data curation. **Gabriela Cavalcanti Lima Albuquerque:** writing – review and editing. **Michelle Melgarejo da Rosa:** writing – review and editing, translation. **Jamile Taniele‐Silva:** writing – review and editing, methodology, investigation, formal analysis, data curation, conceptualization. **Angela Luzia Branco Pinto Duarte:** supervision, project administration, investigation. **Maira Galdino da Rocha Pitta:** supervision, project administration, investigation. **André Machado de Siqueira:** supervision, project administration, investigation. **Amanda Pinheiro de Barros Albuquerque:** writing – review and editing, methodology, investigation, funding acquisition, formal analysis, data curation, conceptualization. **Moacyr Jesus Barreto de Melo Rego:** writing – review and editing, supervision, resources, project administration, methodology, investigation, funding acquisition, formal analysis, data curation.

## Conflicts of Interest

The authors declare that the research was conducted in the absence of any commercial or financial relationships that could be construed as a potential conflict of interest.

## Supporting information


**Figure S1:** Action of CHIKV on mediators across clinical phases of CHIKF and clinical outcomes – Recovered and Persistent Arthralgia after 90 DPSO*, and Control group. A) MMP‐1; B) MMP‐2; C) MMP‐3; D) MMP‐9; E) MMP‐14; F) TIMP‐1. **Figure S2:** Discriminative ability of MMPs and TIMPs for CHIKF clinical outcomes assessed by ROC Curve AUC analysis. A) MMP‐1; B) MMP‐2; C) MMP‐3; D) MMP‐9; E) MMP‐14; F) TIMP‐1; G) TIMP‐2. **Table S1:** Variables with musculoskeletal impact selected from bivariate logistic regression among individuals recruited during the post‐acute phase of the disease.

## Data Availability

The data that support the findings of this study are available from the corresponding author upon reasonable request.
