# Supplementary material for: TIMP‐2 as a Potential Indicator of Persistent Arthralgia in Chikungunya: Evidence From a Brazilian Cohort Study
Source: J Med Virol. 2025 Oct 14;97(10):e70635. doi: 10.1002/jmv.70635 (PMC12519943; doi:10.1002/jmv.70635)

**Supplementary Material**

Table 1 – Variables with musculoskeletal impact selected from bivariate logistic regression among individuals recruited during the post-acute phase of the disease.

| **Variables** | **Crude Bivariate Analysis**  **OR (95% CI); p-value** |
| --- | --- |
| Gender | 2.07 (0.788–5.480); 0.139 |
| Blurred vision | 2.28 (0.732–7.129); 0.154 |
| Paresthesia | 2.35 (1.072–5.162); 0.033 |
| Edema | 2.02 (0.827–4.931); 0.122 |
| Myalgia | 2.04 (0.890–4.690); 0.092 |
| Skin rash | 3.29 (1.213–8.968); 0.019 |
| Morning stiffness | 2.93 (1.133–7.616); 0.027 |
| Joint pain VAS >7 | 2.33 (1.050–5.206); 0.037 |
| Disease activity category | 2.33 (1.050–5.206); 0.037 |
| Swollen joints | 3.54 (1.530–8.213); 0.003 |
| MMP-1 | 0.77 (0.520–1.143); 0.196 |
| TIMP-2 | 2.17 (1.223–3.877); 0.008 |

Legend: Variables such as anemia, neoplasia, and pruritus were excluded from the analysis due to collinearity. Headache and insomnia were removed due to low clinical relevance, as they are nonspecific symptoms for Chikungunya. COPD, jaundice, and arthralgia could not be calculated because one of the analyzed groups did not present these symptoms. Data are presented as Odds Ratios (OR) with 95% Confidence Intervals (95% CI).

Figure 1 – Action of CHIKV on mediators across clinical phases of CHIKF and clinical outcomes – Recovered and Persistent Arthralgia after 90 DPSO*, and Control group. A) MMP-1; B) MMP-2; C) MMP-3; D) MMP-9; E) MMP-14; F) TIMP-1.


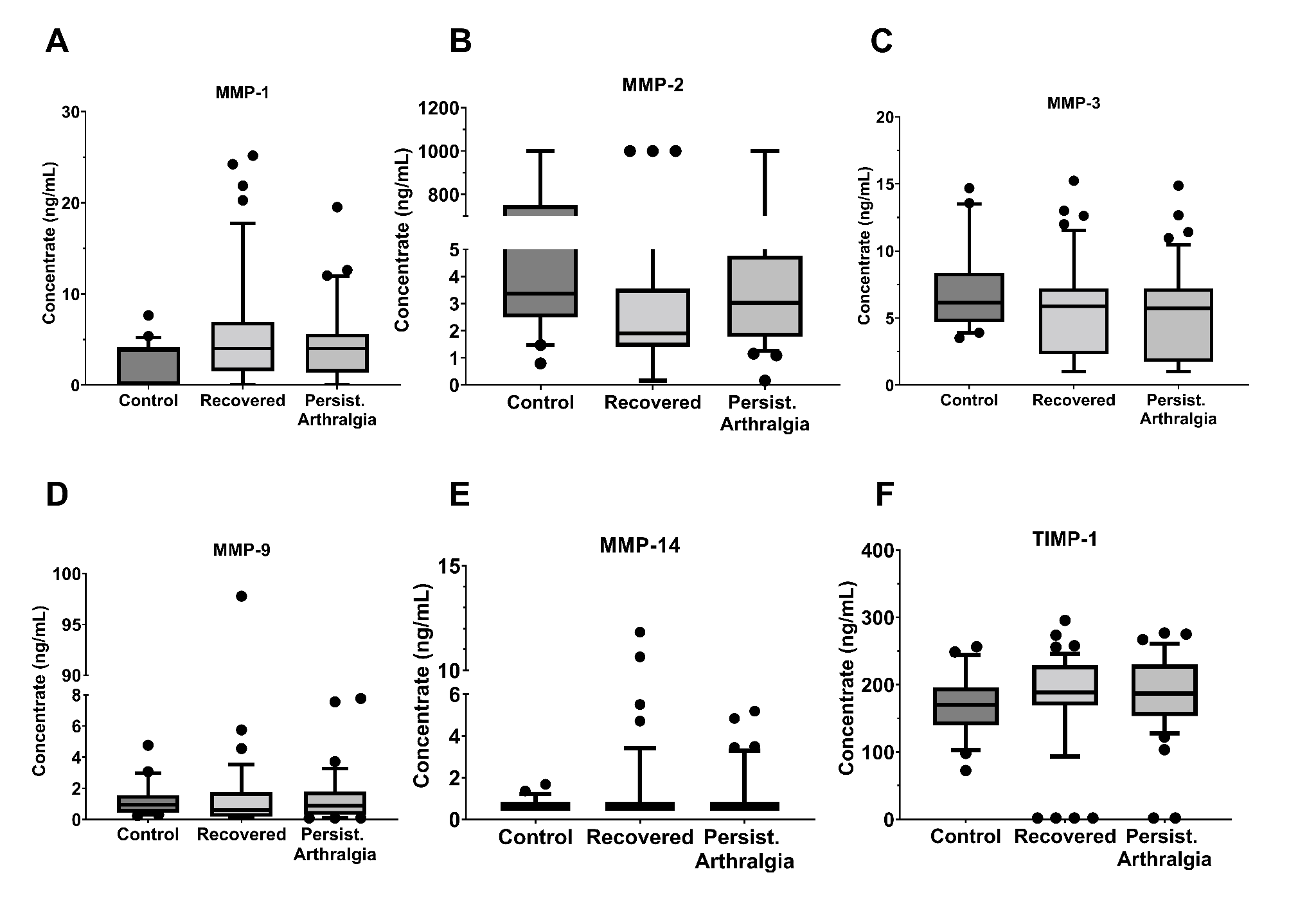


Legend: DPSO = Days Post-Symptom Onset.

Figure 2 – Discriminative ability of MMPs and TIMPs for CHIKF clinical outcomes assessed by ROC Curve AUC analysis. A) MMP-1; B) MMP-2; C) MMP-3; D) MMP-9; E) MMP-14; F) TIMP-1; G) TIMP-2.


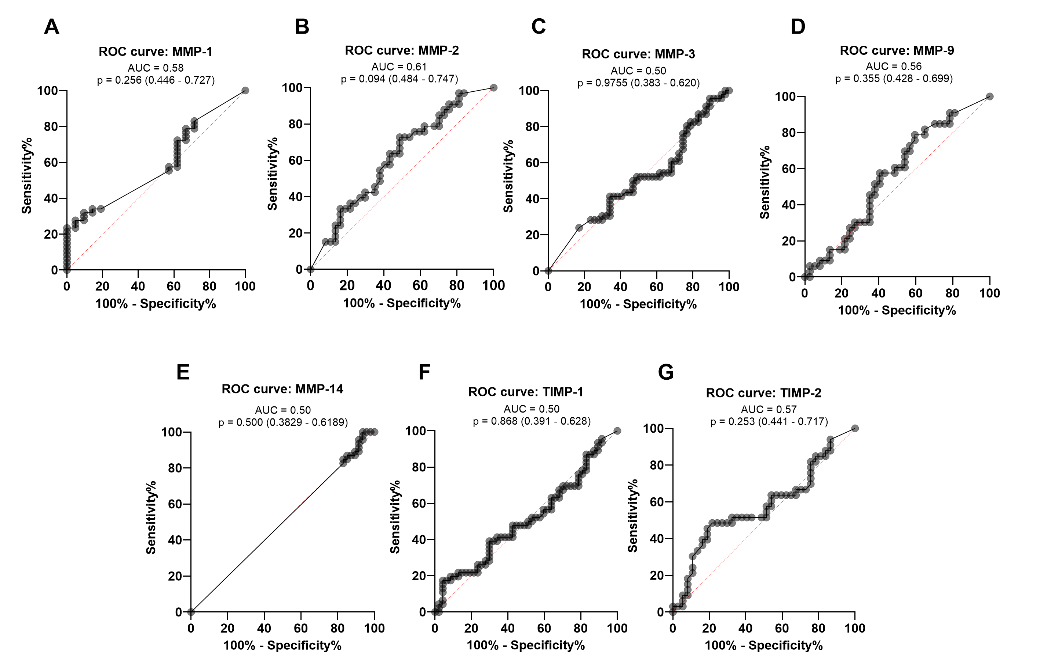

Supplement: Supplementary file 1 — Figure S1: Action of CHIKV on mediators across clinical phases of CHIKF and clinical outcomes – Recovered and Persistent Arthralgia after 90 DPSO*, and Control group. A) MMP‐1; B) MMP‐2; C) MMP‐3; D) MMP‐9; E) MMP‐14; F) TIMP‐1. Figure S2: Discriminative ability of MMPs and TIMPs for CHIKF clinical outcomes assessed by ROC Curve AUC analysis. A) MMP‐1; B) MMP‐2; C) MMP‐3; D) MMP‐9; E) MMP‐14; F) TIMP‐1; G) TIMP‐2. Table S1: Variables with musculoskeletal impact selected from bivariate logistic regression among individuals recruited during the post‐acute phase of the disease. [file JMV-97-e70635-s001.docx]
